# Association between cognitive quotient test score and hippocampal volume: a novel, rapid application-based screening tool

**DOI:** 10.1038/s41598-020-74019-7

**Published:** 2020-10-07

**Authors:** Wataru Kasai, Tadahiro Goto, Yuki Aoyama, Kenji Sato

**Affiliations:** 1 Splink Inc. Japan, Tokyo, Japan; 2 Graduate School of Medical Sciences, University of Fukui, Fukui, Japan; 3 Department of Surgery, Sado General Hospital, Niigata, Japan

**Keywords:** Neuroscience, Cognitive ageing, Cognitive neuroscience

## Abstract

We aimed to develop a brief, preclinical test to screen the reduced hippocampal volume that is a marker of early dementia [Cognitive Quotient (CQ) test]. We performed an observational study of adult subjects who underwent brain MRI in seven institutions from February 2018 to May 2019. The CQ test consists of five components: (1) digits forward, (2) digits backward, (3) Stroop test, (4) simple calculation, and (5) mental rotation. The primary outcome measure was hippocampal volume. We separated the data into derivation (n = 322) and validation cohorts (n = 96). In the derivation cohort, we built two scoring systems using the results of CQ test (model 1 and 2). In the validation cohort, we validated the correlation of the scoring systems with hippocampal volume. In the derivation cohort, there was a moderate correlation between the scoring systems and hippocampal volume [e.g., correlation coefficient = 0.62 in model 1 (95% CI 0.44–0.75)]. Likewise, in the validation cohort, there was a moderate correlation between the scoring systems and hippocampal volume [e.g., correlation coefficient = 0.54 in model 2 (95% CI 0.38–0.67)]. In this analysis of 418 participants, the score of newly developed CQ test was correlated with hippocampal volume.

## Introduction

Dementia is a progressive disorder characterized by a cluster of symptoms and signs, including psychological, psychiatric, cognitive, activity of daily living impairments. Approximately 12 million people worldwide have dementia, and the number will increase to 25 million by 2040^1^. Since the global healthcare burden of dementia is larger than that of stroke, heart disease, and cancer, the benefits of early recognition and interventions are remarkable^2^.

With the increasing use of magnetic resonance imaging (MRI), hippocampal volume has emerged as an attractive marker of early dementia, which was not identified in traditional screening tools, which is often examined as paper-based, due to the ceiling effect^3–7^. In the *National Institute on Aging-Alzheimer’s Association* reported that the pathophysiological changes in medial temporal lobe including hippocampus might be associated with probable Alzheimer disease dementia^8^. However, although the early changes in the hippocampal volume has potential as a marker to detect subclinical dementia^3,4,9,10^, the availability and financial costs of MRI may limit its use as a screening test in the healthy population without any apparent cognitive problems. Therefore, there is a need to develop a brief, preclinical test to screen the reduced hippocampal volume suggesting hippocampal atrophy in the healthy population—a marker of early dementia that is not identified by traditional diagnosis tools due to a ceiling effect in healthy subjects^11^.

To address this concern, we have developed the Cognitive Quotient (CQ) test—a brief, application-based tool on digital devices to screen healthy subjects who may have a small hippocampal volume and require further investigations to detect early dementia. We aimed to examine the relationship between the score of the CQ test (CQ score) and MRI-based hippocampal volume.

## Results

### Characteristics of the participants

From February 2018 to May 2019, a total of 449 participants completed the CQ test and underwent MRI. Among these participants, we excluded 10 participants with acute stroke and 21 participants whose hippocampal volumes could not be successfully abstracted from MRI artifacts. The remaining 418 participants were eligible for the analysis. The median age was 73 years, 53% of the participants were women, 73% had a normal body mass index, 67% had never smoked, and approximately 85% lived with ≥ 1 person (Table 1). Compared with the subjects in the derivation cohort, those in the validation cohort were more likely to be younger and male.

**Table 1:** Patient characteristics in derivation cohort and validation cohort.

	Overall	Derivation cohort	Validation cohort
Variables	n = 418	n = 322	n = 96
Age, median (IQR)	73 (59–80)	75 (66–81)	55 (48–70)
Women	223 (53%)	190 (59%)	33 (34%)
Height, cm, median (IQR)	158 (151–167)	150 (157–165)	157 (166–174)
Weight, kg, median (IQR)	56 (49–65)	55 (48–63)	63 (54–72)
**Body mass index**			
< 18.5	30 (7%)	24 (7%)	6 (6%)
18.5–24.9	305 (73%)	236 (73%)	69 (72%)
25.0–29.9	73 (17%)	55 (17%)	18 (19%)
≥ 30.0	10 (2%)	7 (2%)	3 (3%)
**Education level, years**^**a**^			
6 or 9	38 (9%)	28 (9%)	10 (10%)
12	104 (25%)	73 (23%)	31 (32%)
14	26 (6%)	19 (6%)	7 (7%)
16 or(and) over	77 (18%)	36 (11%)	41 (43%)
N/A	173 (41%)	166 (52%)	7 (7%)
**Smoking history**^**b**^			
Smoking index ≥ 1000	20 (5%)	16 (5%)	4 (4%)
Smoking index < 1000	117 (28%)	79 (25%)	38 (40%)
Never smoker	281 (67%)	227 (70%)	54 (56%)
**Alcohol history**			
Daily	118 (28%)	76 (24%)	42 (44%)
Weekly	73 (17%)	49 (15%)	24 (25%)
Monthly	34 (8%)	30 (9%)	4 (4%)
Not drinking	193 (46%)	167 (52%)	26 (27%)
**Number of persons living with**			
0	57 (14%)	43 (13%)	14 (15%)
1	141 (34%)	113 (35%)	28 (29%)
2	105 (25%)	81 (25%)	24 (25%)
3 or more	115 (28%)	85 (26%)	30 (31%)
**Comorbidity**			
Stroke including subclinical lacunar infarction	35 (8%)	35 (11%)	0 (0%)
Hypertension	52 (12%)	52 (16%)	0 (0%)
Diabetes	35 (8%)	35 (11%)	0 (0%)
Dementia or Alzheimer’s disease	89 (21%)	89 (28%)	0 (0%)
Mild cognitive impairment	11 (3%)	11 (3%)	0 (0%)
Depression	4 (1%)	4 (1%)	0 (0%)
Missing	214 (28%)	118 (37%)	96 (100%)
**Mini-mental status examination**	111 (27%)	111 (34%)	–
0–23	33 (8%)	33 (10%)	–
24–27	34 (8%)	34 (11%)	–
28–30	44 (11%)	44 (14%)	–

Data were expressed as n (%) unless otherwise indicated. Percentages may not equal 100 due to rounding.

*IQR* interquartile range, *N/A* no answer.

^a^Education level was converted to “years of education” for each school category in questionnaire.

^b^Smoking index (Brinkman index) was calculated as “smoking years-x-daily count”.

### Assessment of the CQ score in the derivation cohort

**Figure 1:**
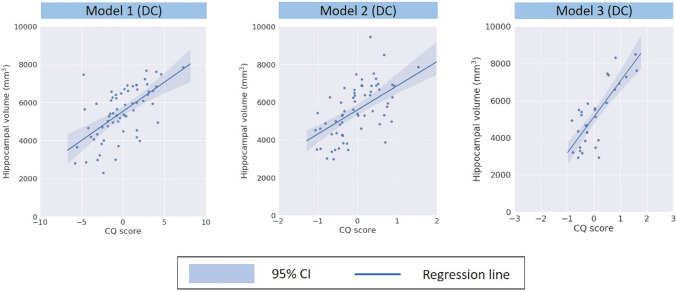
Correlation between Cognitive Quotient (CQ) score and hippocampal volume in the derivation cohort. In the derivation cohort, the model 1 has a moderate correlation between CQ score and hippocampal volume (mean r of the fivefold cross validation, 0.62 [95% CI 0.44–0.75]**)**. Likewise, model 2 has a moderate correlation between CQ score and hippocampal volume (mean r in the fivefold cross validation, 0.61 [95% CI 0.43–0.74]). In the model 3, there was a high correlation between the adjusted CQ score and hippocampal volume (mean r in the fivefold cross-validation, 0.80 [95% CI 0.62–0.90].

In the derivation cohort (n = 322 subjects), the mean CQ score was − 0.19 (standard deviation [SD], 2.72) in model 1 and 0.03 (SD, 0.67) in model 2. The mean hippocampal volume was 5480 mm^3^ (SD, 1354 mm^3^). In the derivation cohort, there was a moderate correlation between the CQ score and hippocampal volume in model 1 (mean r of the fivefold cross-validation, 0.62 [95% CI 0.44–0.75]; Fig. 1, model 1). Likewise, there was a moderate correlation between the CQ score and hippocampal volume in model 2 (mean r in the fivefold cross-validation, 0.61 [95% CI 0.43–0.74]; Fig. 1, model 2). In the model 3, there was a high correlation between the adjusted CQ score and hippocampal volume (mean r in the fivefold cross-validation, 0.80 [95% CI 0.62–0.90]; Fig. 1, model 3). In the graphical assessment, the CQ scores are widely distributed in the high score strata of the MMSE (Supplemental Fig. 2).

### Validation of CQ score

**Figure 2:**
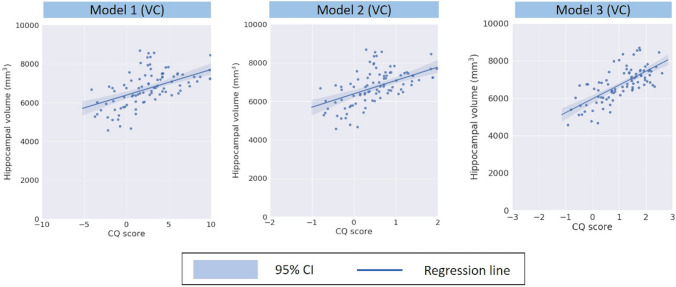
Correlation between Cognitive Quotient (CQ) score and hippocampal volume in the validation cohort. In the validation cohort, the model 1 has a moderate correlation between CQ score and hippocampal volume (r = 0.54 [95% CI 0.38–0.67]**)**. Likewise, the model 2 has moderate correlation between CQ score and hippocampal volume (r = 0.53 [95%CI 0.37–0.66]). In the model 3, there was a high correlation between the adjusted CQ score and hippocampal volume (mean r in the fivefold cross-validation, 0.70 [95% CI 0.62–0.90].

In the validation cohort (n = 96 subjects), the mean CQ score was 2.81 (SD, 3.66) in model 1 and 0.54 (SD, 0.69) in model 2. The mean hippocampal volume was 6758 mm^3^ (SD, 899 mm^3^). In the validation cohort, there was a moderate correlation between the CQ score and hippocampal volume in model 1 (r = 0.54 [95% CI 0.38–0.67]; Fig. 2, model 1). Likewise, there was a moderate correlation between the CQ score and hippocampal volume in model 2 (r = 0.53 [95% CI 0.37–0.66]; Fig. 2, model 2). In the model 3, there was a high correlation between the adjusted CQ score and hippocampal volume (mean r in the fivefold cross-validation, 0.70 [95% CI 0.62–0.90]; Fig. 2, model 3). The detailed of the regression models are shown in Supplemental Table 2**.**

## Discussion

In this study, there was a significant correlation between our newly developed CQ score based on five components of widely-used neuropsychological tests and the hippocampal volume. Furthermore, in the graphical assessment, the CQ scores were widely distributed in the high score strata of the MMSE, suggesting a ceiling effect of the MMSE consistent with our hypothesis that the CQ test could be a potential tool of screening individuals with small hippocampal volume. The strength of our study is the development of digital apps for cognitive testing that was studied with hippocampal volume using MRI.

Early detection of dementia facilitates early interventions that could prevent disease progression and maintain individuals at their current (time of examination) level of cognitive functions^12^. Currently, there are various screening tools developed for early detection of dementia, including the MMSE, the Montreal Cognitive Assessment (MoCA) for mild cognitive impairment detection^13^, and the Clinical Dementia Rating scale (CDR)^14^. However, these tools have been known to possess their own limitations, including their long time taken, patient dependence, and requirement for human resources. The strengths of the CQ test are that it is brief (< 5 min), self-explanatory and intuitive, easy to implement, and relies on objective measurements (i.e., digit forward, and digit backward, Stroop test, simple calculation, mental rotation test) based on the application on digital devices. While the correlation between the CQ score and hippocampal volume was moderate, a high accuracy might be a trade-off for simplicity. Additionally, the aim of the CQ test was not to diagnose dementia but to screen individuals who may have a small hippocampal volume and who may require further examinations (e.g., MRI) to detect early dementia.

The conventional assessment tools represented by the MMSE and the MoCA have been criticized as having a ceiling effect in the very early stage of dementia or subclinical dementia^15,16^. Instead, a small hippocampal volume has been reported as an early sign of dementia prior to clinical symptoms. Multiple studies have reported that a small hippocampal volume was found not only in 80–90% of patients with Alzheimer’s disease^17–20^ but also in patients with mild cognitive impairment^21^, frontotemporal dementia^22^, and vascular dementia^23^. In addition, a small hippocampal volume is associated with the risk of future Alzheimer disease^24^. A 10-year follow-up study on 518 elderly patients found that a majority of the patients who developed dementia had a smaller baseline hippocampal volume years before their clinical diagnosis compared with those who remained dementia-free^3^. These findings collectively indicate that the subtle delayed memory decline with hippocampal volume atrophy can be observed long before a clinical diagnosis of dementia is made^3^. Despite the promising ability of hippocampal volume as a marker of early dementia, the hippocampal volume can only be evaluated using imaging examinations (e.g., MRI), which require financial costs and have limited availability. Thus, as a brief, inexpensive screening tool, the CQ test could be beneficial in identifying patients who need MRI to determine the presence of hippocampal atrophy that is not captured by the MMSE or other traditional screening tools that use the MMSE as a gold standard.

While formal validation is needed, by using the CQ test, physicians can easily screen people with seemingly normal cognitive function for early dementia without cost or time. In addition, because the CQ test is easy to use, people can repeat the test, and it is expected to be able to detect early cognitive decline from not only point estimates but also longitudinal changes of the test score. Lastly, in this study, we focused on the CQ test itself, while the prediction ability should be improved with the use of further characteristics information, such as smoking history, family history, comorbidities, and physical activities, with the use of machine-learning or artificial neural networks. The advantages of the application-based test is scalability; therefore, the development of CQ test as the first step should be an important basis for developing the optimal screening test for early dementia.

### Potential limitations

Our study has several potential limitations. First, although there was a correlation between the CQ score and the hippocampal volume, it does not directly indicate early dementia. However, the primary objective of the CQ test is to screen individuals who need further investigations including high-cost examinations. Second, the study population included participants who underwent MRI for medical reasons, which might have affected cognitive function. However, the correlation between the CQ score and hippocampal volume remained significant in the cohort lacking any healthy participants (i.e., those that underwent MRI for medical check-up reasons). Third, patients in the validation cohort were assumed to be healthy population, there was no neuropsychological evaluation including MMSE. Nevertheless, there were no report on the cognitive problems and no specific findings in their MRI. Fourth, the proposed application-based test lacked the subtest that explicitly evaluates memory domain. This is because word recall, which is the widely used test to measure memory domain, is not well suited for an application designed to be completed in a short time. Thus, there may be room for improvement to the battery by adding an alternate subtest, such as FCSRT^25,26^. Finally, there were no longitudinal data to evaluate the decline rate of the hippocampal volume because this study was based on a single measurement point.

## Conclusions

In this analysis of 418 participants, our newly developed CQ score was significantly correlated with hippocampal volume. Our findings indicate that the CQ test could be a potential tool of screening individuals with small hippocampal volume, which is a marker of mild or subclinical cognitive impairment that cannot be detected by traditional screening tools. While further studies are warranted, this brief, inexpensive, application-based tool could be beneficial in identifying patients at-risk of cognitive problems that may warrant further investigation; not just for MRI but for other diagnostics, including a full neuropsychological evaluation, or other biomarkers.

## Methods

### Study design and setting

We performed an observational study of subjects who underwent brain MRI in seven institutions (four general hospitals and three clinics) from February 2018 to May 2019. There are approximately 30,000–320,000 annual outpatients in the seven institutions. This study was approved by the Institutional Review Board of Sado General Hospital. Written informed consents were obtained from all participants. This study on humans was carried out in accordance with Declaration of Helsinki.

### Study participants

We included adult individuals (aged ≥ 18 years) who underwent brain MRI for a routine checkup or any medical reasons, such as a headache, dizziness, numbness, and medical follow up. When a physician decided to take a brain MRI, the physician also obtained written informed consent and performed a CQ test on the subject. The subject completed the CQ test before receiving the MRI findings. We excluded participants who were not independent, diagnosed with stroke, or whose hippocampal volume could not be successfully abstracted from the MRI results.

### Development of the CQ test

**Figure 3:**
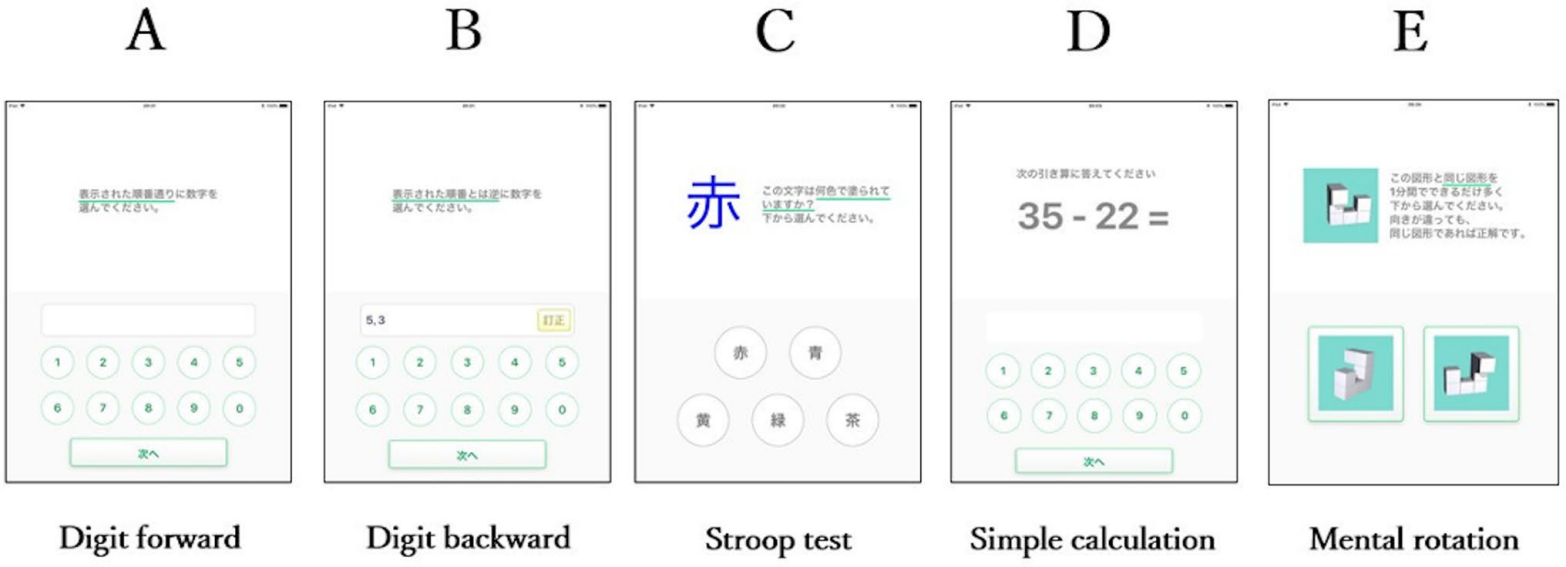
Application format and five questionnaires of the Cognitive Quotient (CQ) test. Panel (**A**) Digit forward test, Panel (**B**) Digit backward test, Panel (**C**) Stroop test, Panel (**D**) Simple calculation test, Paned (**E**) Mental rotation test.

We developed the CQ test as a screening tool for evaluating hippocampal volume to screen individuals who need further investigation for dementia. The CQ test consists of five components based on well-validated and widely-used neuropsychological tests^27–34^. The five components are (1) digits forward (Fig. 3A), (2) digits backward (Fig. 3B), (3) Stroop test (Fig. 3C), (4) simple calculation (Fig. 3D), and (5) mental rotation (Fig. 3E).

Before conducting the current analysis, we conducted a pilot study to identify the optimal set of tests from September 2017 to November 2017. In the pilot study of 36 subjects, we used following test sets based on a priori knowledge: (1) digits forward, (2) digits backward, (3) Stroop test, (4) Addition, (5) Subtraction, (6) N-back task, and (7) delayed recall. Based on the results and correlations between tests, we integrated N-back, delayed recall tests into simple calculation test (i.e., addition and subtraction) because (1) N-back test without test volume limitation to avoid ceiling effect and delayed recall test were time-consuming and had limited feasibility for an application-based test, (2) N-back test was highly correlated with simple calculation (r = 0.43 with addition, r = 0.16 with subtraction), (3) and the correlation coefficient with the hippocampal volume decreased in the case of remaining test set including delayed recall (Supplemental Table 1). Thus, we first applied the following test: (1) digits forward, (2) digits backward, (3) Stroop test, (4) Simple calculation. By focusing on these four tests, testing time has been shortened, and therefore we have added mental rotation test according to the NIA-AA measurements. Consequently, the CQ test consists of (1) digits forward, (2) digits backward, (3) Stroop test, (4) Simple calculation, and (5) mental rotation, and these tests were consistent with the NIA-AA test except for vocabulary test (vocabulary test may be not feasible for an application-based test).

The first and second components are based on the widely-used cognitive test^35,36^. The digits forward and digits backward tests are used to examine the function of working memory, which is related to the medial occipital cortex, dorsolateral prefrontal cortex, bilateral inferior parietal lobule, anterior cingulate, and Broca’s area^37^. For example, there are significant positive correlations between the gray matter ratio, the percentage of gray matter volume in the intracranial volume, and performance on the Digit Span subtest^34^. The third component, the Stroop test, is used to examine the cognitive regulation function and cognitive-related control of dorsolateral prefrontal and anterior cingulate cortex activity involving the hippocampus^27,29,38^. This neuropsychological test is extensively used to assess the ability to inhibit cognitive interference that occurs when the processing of a specific stimulus feature impedes the simultaneous processing of a second stimulus attribute^27^. The fourth component, simple calculation, is used to examine functions related to the bilateral premotor, posterior parietal, and prefrontal cortex^32^. A simple calculation is performed in assessment of prefrontal to posterior parietal cortex activation involving working memory^33,39^. The fifth component, mental rotation of three-dimensional objects test, is used to examine hippocampal-related dorsal and ventral premotor cortex functions^30^. Mental rotation has been described as a rotary transformation of a visual stimulus allowing it to be represented in a new orientation^31^. The CQ score can be calculated based on the results of these five questionnaires. We designed the CQ scoring system to evaluate where the total cognitive score is positioning on standard normal distribution of population. The total CQ score is derived as non-weighted (or weighted) sum of each test score also evaluated on normal distribution for each.

### Main outcomes and measurements

The primary outcome measure was hippocampal volume (in mm^3^), which was measured by an automated-segmentation method from brain MRI as previously described (Supplemental Fig. 1)^3,40^. We collected information of patient demographics, including age, sex, body mass index, smoking history, alcohol use, and the number of persons living with the subject. The MRI findings were reported by board-certified neurosurgeons, neurologists, or radiologists. The primary diagnosis at the MRI examination was abstracted from medical records when the subject had any disease or abnormality based on the physician’s discretion.

### Statistical analysis

We separated the data into derivation and validation cohorts. First, to develop the CQ scoring systems, we used data from six institutions (n = 322, 77% of the overall cohort) as the derivation cohort. The scoring systems were developed using following items: (1) for digit forward and backward: the maximum number of successfully-answered digits; (2) for simple calculation and the Stroop test: the number of correct answers and the mean time to answer, (3) for the mental rotation: the number of correct answers. Next, we built three scoring systems according to the previous literature^41^: (1) the sum of each test scores on standardized normal distribution (model 1), (2) multivariable linear regression model using each component (model 2), (3) and multivariable linear regression model including age and educational level. A is a set of 5 cognitive test set, and x is test score and $\mu$ and σ are mean and standard deviation for each. z(x, $\mu$, σ) is a normal distribution of mean 0 and standard deviation 1 after the standardization.

Model 1

$$CQ=\sum_{i \in A}z\left({x}_{i},{\upmu }_{i},{\upsigma }_{i}\right)$$

Model 2

$$CQ=\sum_{i \in A}{a}_{i}z\left({x}_{i},{\upmu }_{i},{\upsigma }_{i}\right)$$

Model 3

$$CQ=\sum_{i \in A}{a}_{i}z\left({x}_{i},{\upmu }_{i},{\upsigma }_{i}\right)+\sum_{j \in \{age, education\}}{b}_{j}z\left({x}_{j},{\upmu }_{j},{\upsigma }_{j}\right)$$

After developing these scoring systems, we used fivefold cross-validation of the derivation cohort to examine the models’ performance of the models. In the validation cohort using data from a clinic (n = 96, 23% of the overall cohort), we examined the association between the developed CQ scoring systems and the actual hippocampal volume. In the validation cohort, all subjects had undergone MRI for a medical checkup without any symptoms (i.e., healthy population), since the development of our screening tool aimed to identify early dementia that was not identified in the conventional screening tools due to ceiling effect. In addition, we also graphically-assessed the distribution and association between the CQ score and MMSE by using scatter-plot in the derivation cohort. For MRI data, we used Hippodeep^40^ on Mindboggle^42^ as an alternative tool of FreeSurfer^43^ which takes over 10 h for pre-processing to extract the hippocampal volume. Hippodeep is a python-based hippocampal region extraction tool and its processing time is very short (less than 1 min). Moreover, we analyzed data using python 3.5.6 and SciPy library (1.0.0) on Google Datalab (vCPU × 1, 3.75 GB Memory) platform.

## Supplementary information

Supplementary file1

## Data Availability

The datasets generated during and/or analyzed during the current study are not publicly available due to no IRB’s approval of data sharing.
