# Supplementary material for: Association between cognitive quotient test score and hippocampal volume: a novel, rapid application-based screening tool
Source: Sci Rep. 2020 Oct 7;10:16728. doi: 10.1038/s41598-020-74019-7 (PMC7542449; doi:10.1038/s41598-020-74019-7)
Supplement: Supplementary file 1 — Supplementary file1 [file 41598_2020_74019_MOESM1_ESM.docx]

**Association between Cognitive Quotient test score and hippocampal volume: A novel, rapid application-based screening toolAuthors:** Wataru Kasai, PhD; Tadahiro Goto, MD, MPH; Yuki Aoyama, MBA; Kenji Sato, MD

**Supplemental Figure 1. Examples of hippocampal volume calculation in automated-segmentation method from brain MRI**

The colored parts are hippocampal in brain MRI

**Supplemental Figure 2. Graphical assessment of the relation between Cognitive Quotient (CQ) score and Mini-Mental State Examination (MMSE) score in the derivation cohort.**

**Supplemental Table 1. Top 10 combination of subtests correlated with hippocampal volume in the pilot study.**

**Supplemental Table 2. The details of the regression models (model2 and model 3) in the validation cohort**

**Supplemental Figure 1.**

**
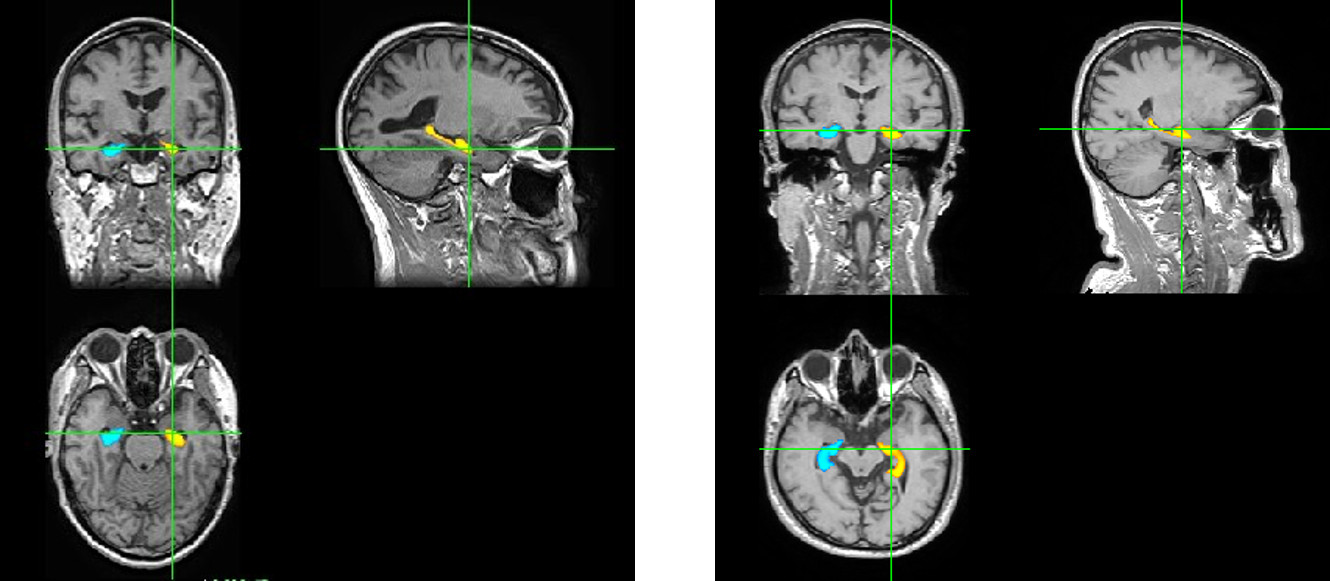
**

**Supplemental Figure 2.**

**
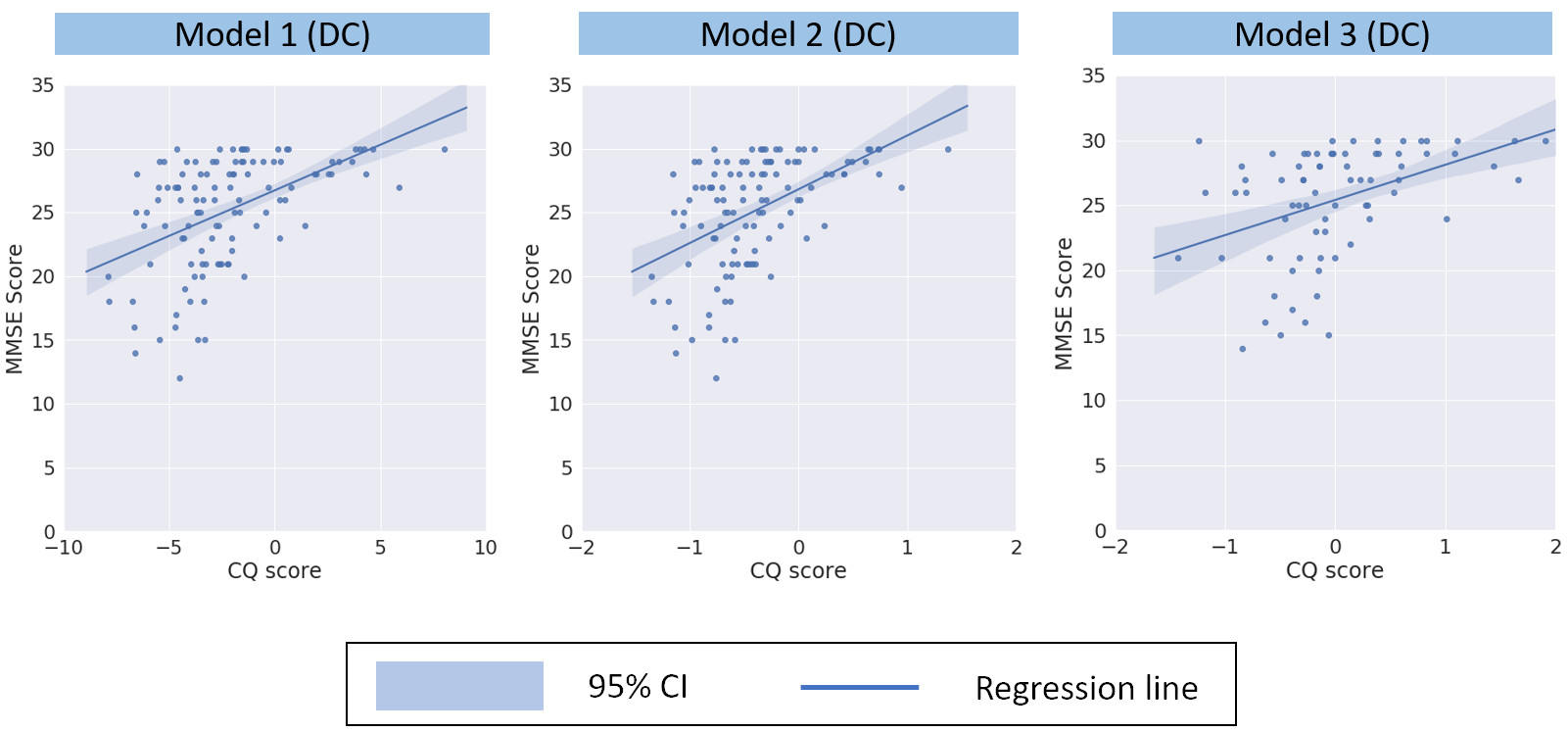
**

**Supplemental Table 1. Top 10 combination of subtests correlated with hippocampal volume in the pilot study.**

| **Ranking** | **Set of subtests** | **Correlation coefficient** | **p-value** |
| --- | --- | --- | --- |
| 1 | Digit forward, Digit backward | 0.18 | 0.30 |
| 2 | Digit forward, Digit backward, Stroop | 0.16 | 0.35 |
| 3 | Digit forward, Digit backward,  Delayed recall | 0.15 | 0.38 |
| 4 | Digit backward | 0.15 | 0.39 |
| 5 | Digit forward, Digit backward, Stroop, Delayed recall | 0.14 | 0.42 |
| 6 | Digit forward | 0.14 | 0.43 |
| 7 | Digit backward, Stroop | 0.13 | 0.44 |
| 8 | Digit backward, Stroop, Delayed recall | 0.12 | 0.47 |
| 9 | Digit backward, Delayed recall | 0.09 | 0.61 |
| 10 | Digit backward, Stroop, Delayed recall | 0.08 | 0.63 |

**Supplemental Table 2. The details of the regression models (model2 and model 3) in the validation cohort**

| **Variables** | **Coefficients** | **Correlation coefficient** | **R2** | **p-value** |
| --- | --- | --- | --- | --- |
| Model 1 |  | 0.53 | 0.28 | < 0.001 |
| Digit forward | 0.05 |  |  |  |
| Digit backward | 0.20 |  |  |  |
| Stroop test | 0.20 |  |  |  |
| Simple calculation | 0.21 |  |  |  |
| Mental rotation | 0.16 |  |  |  |
| Model 1 |  | 0.70 | 0.49 | < 0.001 |
| Digit forward | -0.15 |  |  |  |
| Digit backward | 0.11 |  |  |  |
| Stroop test | 0.01 |  |  |  |
| Simple calculation | 0.37 |  |  |  |
| Mental rotation | 0.01 |  |  |  |
| Age | -0.59 |  |  |  |
| Education | 0.04 |  |  |  |
| Abbreviations: R2: coefficient of determination; | | |  |  |
